# Prolonged Impaired Consciousness and Periodic Sharp Wave Complexes on Electroencephalogram in an Elderly Female With Severe Lithium Intoxication

**DOI:** 10.7759/cureus.93861

**Published:** 2025-10-05

**Authors:** Satoru Ueda, Naofumi Higuchi, Takeshi Inagaki

**Affiliations:** 1 Department of General Internal Medicine, Center Hospital of National Center for Global Health and Medicine, Tokyo, JPN

**Keywords:** bipolar disorder (bpd), electroencephalography (eeg), lithium carbonate, lithium intoxication, lithium side effects, periodic sharp wave complexes

## Abstract

Lithium is commonly used in bipolar disorder but carries a high risk of neurotoxicity. We present a 79-year-old female on lithium for several years who was admitted with fever and altered consciousness that was initially complicated by a urinary tract infection. Based on persistent symptoms and medical history, the patient was found to have a serum lithium level of 3.96 mEq/L (therapeutic range: 0.60-1.20 mEq/L). Despite urgent hemodialysis, her deep coma persisted for nearly two weeks after serum lithium concentration normalized. During the admission course, an electroencephalogram (EEG) revealed periodic sharp wave complexes. This case highlights the importance of maintaining a high index of suspicion for lithium toxicity in bipolar patients with altered mental status and the necessity of supportive care as clinical recovery may be delayed despite normalization of laboratory values.

## Introduction

Lithium has been a standard in the treatment of bipolar disorders for many years. However, it has a narrow therapeutic index and requires close monitoring in order not to cause toxicity. Specifically, the incidence of moderate-to-severe lithium intoxication was 1/100 patient-years, and it is said that advanced age (≥65 years) and impaired renal clearance are well-established risk factors for lithium toxicity [1,2]. It is known that lithium toxicity is a treatable clinical condition that can manifest various neurological symptoms. The clinical severity often correlates with serum lithium levels, and serum levels exceeding 3.5 mEq/L are associated with a serious and potentially life-threatening toxicity [3]. Lithium intoxication can also be classified into three types: acute, acute-on-chronic, and chronic [4]. Acute toxicity generally occurs with the ingestion of a large amount of lithium in a one-time overdose or incidental ingestion. Acute-on-chronic toxicity is seen when a patient who is already receiving chronic lithium therapy takes an acute overdose or has an event that leads to a rapid rise in serum level [5]. Chronic toxicity results from the slow accumulation of lithium secondary to factors impairing lithium clearance, such as dehydration or renal impairment in patients under lithium therapy for the long term.

Clinically, lithium toxicity develops a wide array of symptoms and mainly affects the gastrointestinal and the central nervous systems. Involvement of the central nervous system presents as tremors, incoordination, or more serious complications such as seizures, confusion/altered mental states, and coma [6]. While the majority of neurological symptoms related to lithium toxicity are reversible, a few patients develop chronic deficits [7]. The management is the removal of the lithium from the body, supportive care with fluid resuscitation, and hemodialysis in more severe cases [8]. The recovery time is also variable, such that some patients develop chronic neurological symptoms even after the serum lithium levels return to the normal value [9]. Although the presentation is typically nonspecific, chronic lithium toxicity is more frequently associated with abnormal EEG findings, such as diffuse slowing, triphasic waves, generalized spike and wave, or certain patterns resembling Creutzfeldt-Jakob disease [10-13].

Herein, we report a case of chronic lithium toxicity presenting prolonged unconsciousness and periodic sharp wave complexes in the EEG of an elderly patient to emphasize the importance of unwavering work-up and management despite delay in recovery.

## Case presentation

The patient was a 79-year-old woman with a four-year history of bipolar disorder diagnosed at a local psychiatric clinic, which was precipitated by the death of a relative. Until three weeks before admission, the patient had been managing her daily life well. However, as the anniversary of her relative’s death approached, she refused to eat and could barely drink water. Two weeks before admission, the patient stopped taking her prescribed medications. A week before admission, the patient was taken to the emergency room of another hospital and discharged with a diagnosis of mild dehydration.

Despite her family’s assistance in taking her prescribed medication upon returning home, her speech progressively diminished, and her responsiveness declined. The day before admission, she developed urinary retention and a high fever of 40 degrees Celsius, leading to her presentation at our hospital’s emergency department the next day. Her medical history included bipolar disorder and fibromyalgia. Her regular medications were lithium carbonate, quetiapine, and valproic acid.

In the emergency department, her vital signs were as follows: Glasgow Coma Scale (GCS) score of 7, temperature of 39.6 degrees Celsius, heart rate of 63 beats/minute, oxygen saturation of 94% on 2 L/min of oxygen, and blood pressure of 119/55 mmHg (Table 1). The physical examination revealed no costovertebral angle or abdominal tenderness. There was a marked decrease in muscle tone in both her upper and lower extremities. No gaze deviation or involuntary movements were observed.

**Table 1:** Serial changes in laboratory and clinical findings during hospitalization This table shows the serial changes in the patient's laboratory findings and clinical condition during hospitalization up to day 21. After this point, the patient’s condition stabilized, and subsequent laboratory results remained stable until discharge over a month later. Abbreviations: CRP, C-reactive protein; BUN, blood urea nitrogen; eGFR, estimated glomerular filtration rate; BG, Blood Glucose; WBC, white blood cell; Hb, hemoglobin; PLT, platelet; GCS, Glasgow Coma Scale; O2, oxygen; RA, Room Air

Parameter	Reference range	On admission (Day 1)	Day 8	Day 13	Day 21
Laboratory Findings					
Lithium concentration (mEq/L)	0.6-1.2	3.96	0.10	<0.01	-
CRP (mg/dL)	<0.14	3.93	0.79	0.24	0.33
BUN (mg/dL)	8.0-20.0	88.0	10.4	11.5	12.7
Creatinine (mg/dL)	0.46-0.79	3.17	0.49	0.44	0.41
eGFR (mL/min/1.73 m^2^)		11.6	89.3	100.4	108.5
Sodium (mEq/L)	138-145	143	139	137	144
Potassium (mEq/L)	3.6-4.8	4.8	4.5	4.4	3.1
BG (mg/dL)	73-109	104	132	166	123
WBC count (/μL)	3,300-8,600	18,310	13,580	9,960	6,540
Hb (g/dL)	11.6-14.8	14.0	12.2	12.0	11.5
PLT count (× 10^4^ /μL)	15.8-34.8	18.1	15.9	45.2	21.8
Vital Signs					
GCS level		E1V2M4	E1V2M4	E4V3M4	E4V4M6
Body Temperature (℃)		39.6	36.7	36.3	36.4
Blood Pressure (mmHg)		119/55	135/72	126/67	114/61
Pulse Rate(bpm/min)		63	72	75	69
Oxygen Saturation (%)		94 (on 2L/min O_2)_	96 (RA)	97 (RA)	98 (RA)

Laboratory tests revealed a blood urea nitrogen (BUN) level of 88.0 mg/dL (normal range: 8.0-20.0 mg/dL), serum creatinine level of 3.17 mg/dL (normal range: 0.46-0.79 mg/dL), white blood cell count of 18,310/μL (normal range: 3,300-8,600 /μL), and C-reactive protein (CRP) level of 3.93 mg/dL (normal range: 0.0-0.14 mg/dL). Her estimated glomerular filtration rate (eGFR) was 11.6 mL/min/1.73 m^2^. The serial changes in these key laboratory parameters and clinical condition are detailed in Table 1. Urinalysis indicated bacteriuria and pyuria. Cerebrospinal fluid examination was normal.

After admission, the urinary tract infection was suspected to have led to a transient disturbance of consciousness, and ceftriaxone was initiated. Additionally, psychological stress-induced stupor was considered as a possible diagnosis given her history of bipolar disorder. However, her impaired consciousness persisted for several days despite the initiation of treatment for the urinary tract infection, resolution of her fever, and improvement in inflammatory markers.

**Figure 1:**
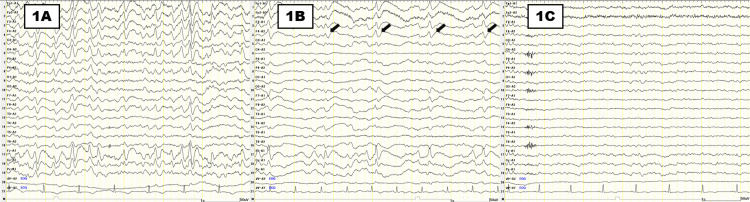
Sequential changes in the EEG along the clinical course (1A) EEG demonstrating the bursts of generalized sharp waves on the fifth day. (1B) EEG showing periodic sharp wave complexes (solid arrows) occurring at approximately 2 Hz intervals on the eighth day. (1C) EEG revealing subsequent normalization of background activity, characterized by posterior-dominant alpha rhythm at 8-10 Hz on the 15th day. EEG filter settings: low-frequency filter at 1.0 Hz, high-frequency filter at 50 Hz, and a 50 Hz notch filter; sensitivity, 10 µV/mm

On the fifth day of hospitalization, the patient developed mouthing-like involuntary movements, indicating orofacial dyskinesia, and an EEG detected bursts of generalized sharp wave (Figure 1A). On the same day, her serum lithium level at the time of admission was found to be 3.96 mEq/L (therapeutic range: 0.60-1.20 mEq/L). Suspecting that chronic lithium intoxication caused her prolonged disturbance of consciousness, she underwent hemodialysis on the sixth and seventh days of her hospital stay.

**Figure 2:**
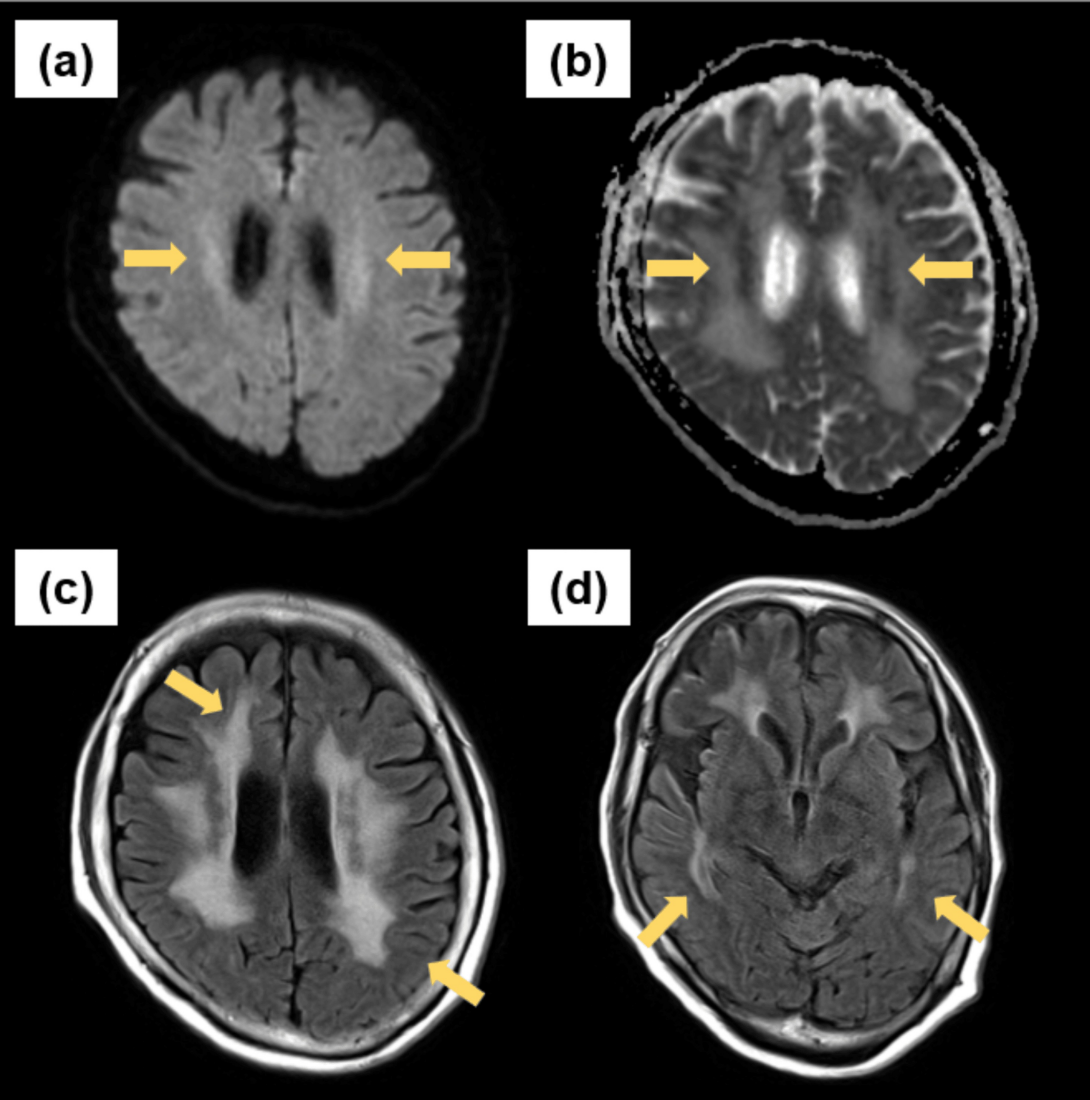
Axial MRI obtained on the 12th day (a) DWI demonstrated subtle hyperintense signals within the bilateral centrum semiovale (arrows). (b) Corresponding ADC map revealed hypointense areas, indicative of restricted diffusion (arrows). (c) Axial T2-FLAIR showed hyperintense lesions in the deep white matter (top arrow) with sparing of the subcortical U-fibers (bottom arrow). (d) Additional T2-FLAIR slice demonstrated bilateral temporal lobe hyperintensities (arrows).

After two hemodialysis sessions, the patient’s serum lithium level decreased to 0.10 mEq/L by the eighth day; her EEG showed periodic sharp wave complexes occurring at approximately 2 Hz intervals (Figure 1B). An MRI on the 12th day revealed faint hyperintensities in the centrum semiovale on diffusion-weighted imaging (DWI), while the apparent diffusion coefficient (ADC) map showed hypointense lesions. T2-weighted fluid-attenuated inversion recovery (T2-FLAIR) showed symmetric hyperintense lesions involving the deep cerebral white matter, as well as bilateral temporal lobes. These findings are consistent with a chronic white matter encephalopathy (Figure 2).

Although her level of consciousness did not show immediate improvement, she recovered to a GCS score of 11 (E4V3M4) on the 13th day and was able to state her name. A repeat EEG performed on the 15th day was normal with no evidence of epileptic discharges (Figure 1C). By the 21st day, her GCS score had improved to 14 (E4V4M6), and she was able to engage in simple conversation and eat independently. Her muscle tone also gradually improved. No recurrence of consciousness disturbance occurred. She made good progress with rehabilitation and was transferred to another facility for continued rehabilitation on the 61st day, at which time she was able to walk with a cane.

## Discussion

The current case provides a few important lessons, which are described below: prolonged impairment of consciousness can occur in chronic lithium toxicity, even in the setting of serum normalization, and EEG abnormalities can be seen in the pattern of periodic sharp wave complexes.

Firstly, it is important to recognize that prolonged impairment of consciousness has been observed even when serum lithium concentrations fall to normal. In the most severe cases, a critical condition referred to as the syndrome of irreversible lithium-effectuated neurotoxicity (SILENT) may occur, and neurological dysfunctions persist for several months to years, occasionally progressing to irreversible deficits [9]. The underlying mechanisms of lithium neurotoxicity are multifactorial, including both biochemical and pharmacokinetic factors. Lithium accumulates not only in the serum but also within cells such as neurons in the central nervous system (CNS) during chronic toxicity. The blood-brain barrier is relatively permeable to lithium ions, but once inside the CNS, the lithium equilibrates slowly between extracellular and intracellular compartments [8,14]. Despite a rapid decrease in serum lithium through hemodialysis, clearance of intracellular lithium remains significantly slower, such that it allows for a rebound phenomenon and persistent neurotoxicity [15]. The continuation of the neurological symptoms is deemed to stem from the delayed elimination of lithium from neural cells; therefore, the cells remain exposed to lithium despite the normalization of serum concentration [9].

The following case presents key points for severe neurotoxicity in chronic lithium toxicity, although no serious symptoms as seen in SILENT have been observed. The patient’s presentation of prolonged impaired consciousness underscores the possibility of a delayed nervous system recovery in lithium toxicity. The diagnosis was established based on orofacial dyskinesia with the abnormal EEG and the markedly elevated serum lithium concentration.

Secondly, EEG abnormalities are a well-documented presentation of lithium poisoning and range from background rhythm changes to seizure-like activity. The most typically reported changes are the diffuse background rhythm slowing and, as the toxicity becomes severe, more prominent abnormalities, such as triphasic waves, rhythmic delta activity, and generalized epileptiform discharges appear [16]. These abnormalities more widely correlate with the degree of clinical neurotoxicity rather than the serum lithium level, due to the fact that they persist after normalization of the serum lithium [13]. Additionally, an important feature of lithium-induced encephalopathy is that the clinical recovery tends to lag behind the restoration of the EEG abnormalities [17]. A previous report has noted that EEG patterns resolve prior to complete recovery of consciousness and thus reflects that electrophysiological normalization does not correlate in turn with the simultaneous clinical improvement [18].

The development of periodic sharp wave complexes in this patient was an important indication of extreme neurotoxicity. Although the serum lithium level returned to normal within a few days, the pathological waveforms remained and recovered in a slow and gradual manner. Alongside this, the sensorium of this patient also improved in a marked delay following the normalization of EEG abnormalities. This delay proves the residual neurotoxicity or delayed recovery of neurons in prolonged encephalopathy.

Neurological recovery from chronic lithium toxicity is typically prolonged, often lasting for weeks even after lithium discontinuation and normalization of serum levels. It is said that the median length of hospital stay is 13 days, mostly due to delayed neurological recovery, and the duration of improvement of neurological symptoms after normalization of lithium concentration is approximately 18 days [19]. However, improvement sometimes extends much longer than that, with some patients taking several weeks to months to achieve full recovery [18,20]. In this case, the prolonged period of impaired consciousness, lasting for nearly two weeks after the normalization of her lithium level, emphasizes the point that clinical resolution may take longer than the improvement seen in laboratory results, which have been influenced by her advanced age and reduced lithium clearance due to renal impairment.

## Conclusions

This case report describes the hemodialysis treatment of a severe case of lithium intoxication in an elderly patient with bipolar disorder. This case reminds clinicians to have a high degree of suspicion for lithium toxicity in patients with bipolar disorder with altered mental status despite other apparent causes. It also reminds us that clinical recovery may take a significant amount of time even after serum lithium levels have been corrected. Additionally, it notifies us that there may be a delay in the full recovery of consciousness even after complete resolution of abnormal EEG patterns. Early diagnosis, vigorous hemodialysis therapy, and supportive care are paramount most important for a successful outcome in such a complicated case.

## References

[1] Ott M, Stegmayr B, Salander Renberg E, Werneke U (2016). Lithium intoxication: incidence, clinical course and renal function - a population-based retrospective cohort study. J Psychopharmacol.

[2] Chan BS, Cheng S, Isoardi KZ (2020). Effect of age on the severity of chronic lithium poisoning. Clin Toxicol (Phila).

[3] Decker BS, Goldfarb DS, Dargan PI (2015). Extracorporeal treatment for lithium poisoning: systematic review and recommendations from the EXTRIP Workgroup. Clin J Am Soc Nephrol.

[4] Murphy N, Redahan L, Lally J (2023). Management of lithium intoxication. BJPsych Adv.

[5] McKnight RF, Adida M, Budge K, Stockton S, Goodwin GM, Geddes JR (2012). Lithium toxicity profile: a systematic review and meta-analysis. Lancet.

[6] Ivkovic A, Stern TA (2014). Lithium-induced neurotoxicity: clinical presentations, pathophysiology, and treatment. Psychosomatics.

[7] Adityanjee Adityanjee, Munshi KR, Thampy A (2005). The syndrome of irreversible lithium-effectuated neurotoxicity. Clin Neuropharmacol.

[8] Baird-Gunning J, Lea-Henry T, Hoegberg LC, Gosselin S, Roberts DM (2017). Lithium poisoning. J Intensive Care Med.

[9] Konieczny K, Detraux J, Bouckaert F (2024). The syndrome of irreversible lithium-effectuated neurotoxicity: a scoping review. Alpha Psychiatry.

[10] Tan HJ, Lim KY, Rajah R, Ng CF (2021). Lithium neurotoxicity with electroencephalogram changes. BMJ Case Rep.

[11] Madhusudhan BK (2014). Nonconvulsive status epilepticus and Creutzfeldt-Jakob-like EEG changes in a case of lithium toxicity. Epilepsy Behav Case Rep.

[12] Amrutha PC, Narayanan Nambiar P, Navaf KM, Aslam S, Krishnadas NC, Pillai DP, Sureshbabu S (2022). New onset encephalopathy with toxic triphasic waves. Int J Epilepsy.

[13] Mucci A, Volpe U, Merlotti E, Bucci P, Galderisi S (2006). Pharmaco-EEG in psychiatry. Clin EEG Neurosci.

[14] Malhi GS, Outhred T (2016). Therapeutic mechanisms of lithium in bipolar disorder: recent advances and current understanding. CNS Drugs.

[15] Jacobsen D, Aasen G, Frederichsen P, Eisenga B (1987). Lithium intoxication: pharmacokinetics during and after terminated hemodialysis in acute intoxications. J Toxicol Clin Toxicol.

[16] Suda M, Kubota F, Aihara Y (2009). A case of lithium intoxication with periodic sharp waves. Pharmacopsychiatry.

[17] Weiner RD, Whanger AD, Erwin CW, Wilson WP (1980). Prolonged confusional state and EEG seizure activity following concurrent ECT and lithium use. Am J Psychiatry.

[18] Jain M, Pokhriyal SC, Chowdhury T (2024). Unraveling enigma of syndrome of irreversible lithium effectuated neurotoxicity: a silent symphony. Crit Care Med.

[19] Hlaing PM, Isoardi KZ, Page CB, Pillans P (2020). Neurotoxicity in chronic lithium poisoning. Intern Med J.

[20] Nambudiri DE, Meyers BS, Young RC (1991). Delayed recovery from lithium neurotoxicity. J Geriatr Psychiatry Neurol.

